# Draft Genome Sequence of Avian Chlamydia abortus Genotype G1 Strain 15-70d24, Isolated from Eurasian Teal in Poland

**DOI:** 10.1128/MRA.00658-19

**Published:** 2019-08-15

**Authors:** Kinga Zaręba-Marchewka, Monika Szymańska-Czerwińska, Agata Mitura, Krzysztof Niemczuk

**Affiliations:** a Department of Cattle and Sheep Diseases, National Veterinary Research Institute, Puławy, Poland; b Laboratory of Serological Diagnosis, National Veterinary Research Institute, Puławy, Poland; University of Maryland School of Medicine

## Abstract

Here, we report the draft genome sequence of avian Chlamydia abortus genotype G1 strain 15-70d24, isolated from Eurasian teal in Poland. The total genome assembly length is 1,149,382 bp.

## ANNOUNCEMENT

Chlamydia spp. are Gram-negative, obligate intracellular bacteria responsible for a broad range of diseases in animals and humans. The genus Chlamydia comprises five candidate species (1–4) and 11 recognized species (5). Chlamydia psittaci, which is encountered mainly in birds, can cause avian chlamydiosis, whereas Chlamydia abortus is the etiological agent of abortion in pregnant ruminants and other mammals (6, 7). Both pathogens represent a zoonotic risk to humans and may lead to significant economic losses worldwide (8, 9). Interestingly, the presence of C. abortus in nonmammalian hosts has also been reported recently (10, 11). The strains originating from wild birds were provisionally named avian *C. abortus*. It seems these strains are epidemiologically relevant, as can be deduced from their worldwide distribution in various bird families (Anatidae, Corvidae, Psittacidae, and Rallidae) (11–13).

Here, we present the draft genome sequence of avian Chlamydia abortus genotype G1 strain 15-70d24, isolated from Eurasian teal in our previous study (11). Taxonomic classification should reflect phylogenetic relationships and avoid the creation of paraphyletic taxa, such as C. psittaci, comprising, according to the current definition, also the C. psittaci/C. abortus intermediates demonstrating features of both C. psittaci and *C. abortus* (11). Therefore, the draft genome sequence of strain 15-70d24 genotype G1 will contribute to the understanding of genetic diversity and the creation of a taxonomic definition of C. abortus, including avian strains, consistent with the current knowledge and updates.

An avian *C. abortus* strain representing genotype G1, 15-70d24, was isolated from a cloacal swab from Eurasian teal on buffalo green monkey (BGM) cell culture and propagated in T25 flasks for 72 h based on procedure published previously (11). After several passages, the cell culture was used for DNA extraction using a QIAamp DNA minikit (Qiagen, Germany), according to the manufacturer’s instructions. DNA was subjected to host-methylated DNA depletion using the NEBNext microbiome DNA enrichment kit (New England BioLabs, USA). Genomic libraries were prepared using the Nextera XT DNA library preparation kit and Nextera XT index kit (Illumina, USA). The DNA concentration of the tested sample was adjusted to 0.2 ng/μl, whereas the total input was 1 ng. Sequencing was conducted on a MiSeq sequencer (Illumina) with the 2 × 300-bp paired-end protocol. Read quality was checked using FastQC 0.1.18 (14), whereas adapters and low-quality sequences were trimmed with Trimmomatic 0.36 (15). Nonchlamydial reads pertaining to the host DNA (from BGM cells) were identified through mapping against the African green monkey genome using BWA-MEM 0.7.15 (16) and removed. The total number of filtered reads was 636,103 (144,572,325 bp). The remaining reads were assembled using SPAdes 3.11.1 (17). Coverage cutoff was set at 10× and a 500-bp length, which yielded 2 contigs. The assembly resulted in scaffold 1 (1,141,702 bp), representing the chromosome, and scaffold 2 (7,680 bp), representing plasmid p70d24. The average read coverages across the chromosome and plasmid were 124× and 140×, respectively. The total genome assembly was 1,149,382 bp long, with a GC content of 39.5%, whereas the *N*_50_ value amounted to 1. Detailed genome properties are presented in Table 1. Default parameters were used for all software programs, unless otherwise specified.

**TABLE 1:** Genome properties of avian Chlamydia abortus genotype G1 strain 15-70d24

Feature^*a*^	Value	% of total
Chromosome		
No. of contigs	1	
Length (bp)	1,141,702	100
Avg read coverage (×)	∼124	
DNA coding (bp)	1,016,691	89.05
DNA G+C content (%)	39.8	
Total no. of genes	1,083	100
No. of:		
Protein-coding genes	1,042	96.21
RNA genes	41	3.79
Pseudogenes	0	
Genes with function prediction	715	66.02
Genes assigned to COGs	499	46.08
Genes with Pfam domains	750	69.25
Genes with signal peptides	56	5.17
Genes with transmembrane helices	244	22.53
CRISPRs	0	
Plasmid		
No. of contigs	1	
Length (bp)	7,680	
Avg read coverage (×)	∼140	
DNA G+C content (%)	33.0	
No. of CDS	8	

a COG, Clusters of Orthologous Groups; CDS, coding sequences.

### Data availability.

The genome sequence of avian Chlamydia abortus strain 15-70d24 genotype G1 has been deposited at the ENA/GenBank/DDBJ under the accession number LS450958 for the chromosome and LS450959 for the plasmid, whereas raw reads have been deposited under the accession number ERS2484026 as a part of BioProject number PRJEB26715.
